# Assessing the Feasibility of Preoperative Axillary Ultrasound in Identifying Node-Negative Axillae: An Indian Retrospective Experience

**DOI:** 10.3390/diagnostics16121874

**Published:** 2026-06-16

**Authors:** Sanika Limaye, Harveen Arora, Rupa Mishra, Mugdha Pai, Nutan Jumle, Namrata Athavale, Chaitanyanand Koppiker, Sneha Joshi, Beenu Varghese

**Affiliations:** 1 Prashanti Cancer Care Mission (PCCM), Pune 411048, India; cra1@prashanticancercare.org (S.L.); rupamishra@prashanticancercare.org (R.M.); namrata.athavale@prashanticancercare.org (N.A.); dr.koppiker@prashanticancercare.org (C.K.); 2 Centre for Translational Cancer Research, Joint Initiative by Indian Institute of Science Education and Research (IISER) Pune and Prashanti Cancer Care Mission, Pune 411048, India; 3 Orchids Breast Health Centre, in Association with PCCM and Jehangir Hospital, Pune 411001, India; radiologythesis@prashanticancercare.org (H.A.); mugdhapai@prashanticancercare.org (M.P.); 4 Jehangir Hospital, Pune 411001, India; nutan.jumle@jehangirhospital.com

**Keywords:** preoperative axillary ultrasound (PAUS), early-stage breast cancer, sentinel lymph node biopsy (SLNB), axillary management, Indian cohort

## Abstract

**Background and Objectives**: Preoperative axillary ultrasound (PAUS) is a non-invasive method to assess nodal metastasis in breast cancer. Although sentinel lymph node biopsy (SLNB) is the gold standard, PAUS may help identify patients who can safely omit SLNB. This study evaluates PAUS’s diagnostic accuracy in predicting axillary nodal negativity (N0) in early-stage breast cancer. **Methods**: This retrospective study included 165 patients with confirmed early-stage breast cancer, excluding those with prior malignancies, neoadjuvant chemotherapy, or palpable axillary lymphadenopathy. PAUS classified nodes as positive or negative using stringent sonographic criteria, and findings were correlated with SLNB histopathology. Accuracy for detecting negative axillae, performance in patients meeting SOUND trial criteria, and overall diagnostic parameters were calculated. **Results**: Of the 165 patients, 86 were identified as node negative on PAUS, with a 90.69% accuracy for detecting negative nodes. For the full cohort, PAUS showed a sensitivity of 86.20%, specificity of 71.02%, positive predictive value of 61.72%, negative predictive value of 90.47%, and overall accuracy of 76.36% for identifying nodal status. Significant nodal features included shape, fatty hilum, and margins (*p* < 0.001), along with primary tumor size (*p* = 0.004). Histopathological findings such as extranodal extension (*p* < 0.001) and lymphovascular invasion (*p* < 0.001) were also significant. **Conclusions**: PAUS demonstrated high accuracy for identifying negative axillae and strong sensitivity and NPV, indicating it may identify node-negative patients who may forgo SLNB. These results support PAUS as a valuable tool for axillary surgery de-escalation, with further prospective validation recommended.

## 1. Introduction

Sentinel lymph node biopsy (SLNB) remains the gold standard for axillary staging in patients with clinically node-negative early-stage breast cancer (ESBC), providing critical prognostic information that guides adjuvant therapy decisions. Axillary lymph node (ALN) status, along with tumor biology, continues to play a key role in determining recurrence risk and overall survival [1]. While much of the Indian population continues to present with advanced-stage disease due to limited organized screening, tertiary referral centers in metropolitan (tier-1) cities are increasingly encountering an earlier-stage presentation and a higher proportion of clinically node-negative disease that may be attributed to relatively better healthcare facilities and general awareness in the urban population [2,3,4].

Although SLNB is less morbid than axillary lymph node dissection (ALND), it remains an invasive staging procedure associated with complications such as lymphedema, sensory disturbances, and restricted shoulder mobility, all of which can significantly impact quality of life. Furthermore, SLNB and ALND are both used for axillary staging in breast cancer, although they differ in roles. SLNB currently is used in ESBC as a minimally invasive staging procedure, while ALND is now mainly reserved for patients with significant nodal disease [5]. Evolving evidence supporting a shift toward less invasive, de-escalated axillary management has led to growing interest in such strategies, particularly in carefully selected low-risk groups [6,7,8,9,10].

At our high-volume tertiary breast cancer center, where 60% of the patients present with node negative disease, SLNB is routinely performed irrespective of preoperative imaging findings, providing a robust histopathological reference standard. In this retrospective observational study, we aim to evaluate the diagnostic accuracy of preoperative axillary ultrasound (PAUS) in identifying node-negative axillae within our setting. Large prospective trials such as SOUND [8] and INSEMA [10] have demonstrated that omission of SLNB in patients with clinically and sonographically node-negative axillae does not compromise oncologic outcomes. However, the generalizability of these findings to heterogeneous healthcare settings, including India, remains uncertain, particularly given variations in imaging expertise and patient presentation. By assessing its performance against final histopathology, this study seeks to determine whether PAUS can reliably identify patients who may be candidates for future de-escalation of axillary surgery, while acknowledging that prospective validation would be required before any change in clinical practice.

## 2. Materials and Methods

### 2.1. Study Design

This is a retrospective observational study with a chart and image review approved by the Institutional Ethics Committee (IEC) that was conducted for early-stage breast cancer patients from a single tertiary breast care center in India from 2022 to 2024 who fulfilled the inclusion and exclusion criteria. Patient consent were not required as only anonymized data was used.

### 2.2. Patient Selection

**Figure 1:**
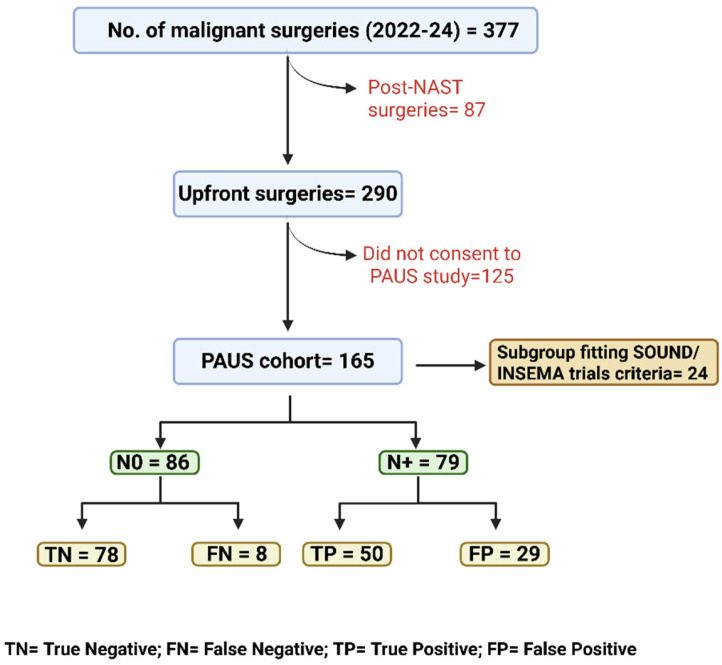
Patient selection and categorization flowchart. Image created in https://BioRender.com.

Figure 1 visualizes the patient selection workflow for the PAUS cohort.

**Inclusion criteria:**

- Any age;
- Tumor staging up to T3;
- Undergoing upfront surgery.

**Exclusion criteria:**

- History of prior malignancy;
- Patients who underwent neoadjuvant chemotherapy (NACT);
- Patients with palpable nodes, inflammatory breast cancer, unknown stage or clinical nodal status, unknown receptor status, clinical T4 or stage IV;
- Patients with psychopathological or addictive disorders or any other compromising conditions.

### 2.3. Data Collection

A cohort of 165 patients fulfilling the inclusion and exclusion criteria were enrolled for this study. All these patients underwent PAUS. US was performed using Philips iU22 (Philips Healthcare, Amsterdam, The Netherlands), Siemens ACUSON S2000 (Siemens Healthcare Private Limited, Issaquah, DC, USA) and Voluson E6 ultrasound machines (GE Healthcare, Zipf, Austria), with the probe frequency set at 6–15 MHz by experienced breast radiologists (each with >5 years of breast imaging experience) with a mean duration of ≈20–30 min per examination. The patient was asked to take an oblique supine position raising the ipsilateral shoulder and placing the arm overhead. Level I and II axillary nodes along with internal mammary nodes were assessed, and if found suspicious, Level III nodes were also examined. The level of the nodes, their number and morphology were noted. The breast radiologists independently reviewed all ultrasound images. Surgical intervention including axillary management was performed for all patients in at median interval of 14 days post-radiological assessment that included PAUS. PAUS findings were then correlated with final histopathological examination (HPE) findings. In cases where reports lacked descriptive details, archived ultrasound images were reviewed again to ensure complete data consistency.

### 2.4. Data Analysis

#### 2.4.1. PAUS Differentiation Criteria

Nodes were distinguished as N0 (negative nodes) and N+ (positive nodes) on PAUS based on the following criteria:

N0: Cortical thickness < 3 mm, intact fatty hilum, oval shape, circumscribed margins.

N+: Cortical thickness ≥ 3 mm, displaced/lost fatty hilum, round shape, non-circumscribed margins.

#### 2.4.2. Evaluation of the Diagnostic Performance of PAUS

PAUS findings were compared to final histopathological results with statistical parameters like sensitivity, specificity, accuracy, positive predictive value (PPV), negative predictive value (NPV), positive likelihood ratio (LR+) and negative likelihood ratio (LR-). Accuracy of PAUS to detect N0 (node negativity) was separately calculated.

#### 2.4.3. Secondary Analyses

The patient’s age, menopausal status, tumor characteristics, clinical axillary status, final pathology, tumor and nodal stages, lymphovascular invasion, planned method for surgery and molecular subtype (ER, PR and HER2 status) were noted. These clinical and HPE findings of SLNB or ALND were used as a reference standard for comparison with the radiological findings. The significance of association between the PAUS features and clinicopathological factors was determined by bivariate analysis with Chi-square (χ^2^) and Fisher’s exact tests.

A sub-group analysis of patients fitting the SOUND and INSEMA trial criteria was done as an observation to understand the accuracy of PAUS at our centre for classifying N0 in those select patients.

## 3. Results

### 3.1. Overview of the Study Cohort

**Figure 2:**
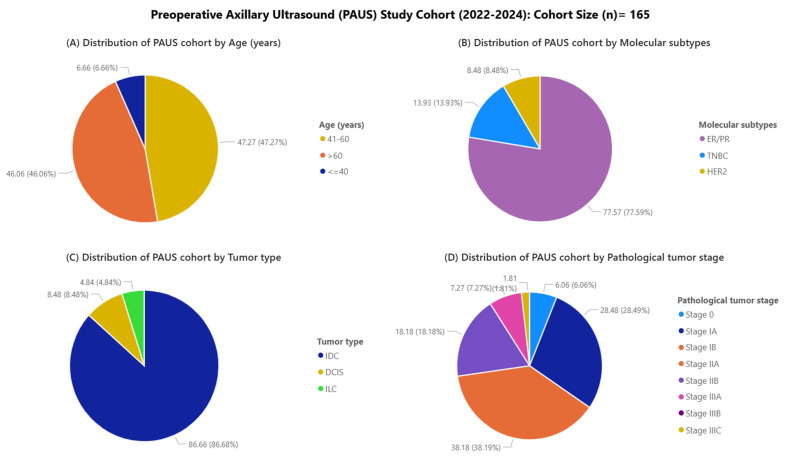
This figure highlights the demographic and clinicopathological distribution for this study cohort (2022–2024). (**A**) Distribution by age. (**B**) Molecular subtype distribution. (**C**) Cohort distribution by tumor type. (**D**) Distribution according to pathological tumor stage (AJCC).

The demographic distribution of the cohort is summarized in Figure 2 and Table 1. This study retrospectively analyzed 165 upfront cases who underwent PAUS from 2022 to 2024. The median age of the patients in the cohort was 60 years (range 27–84 years). About half of the cohort—54.54% (90/165)—had comorbidities like diabetes and hypertension. The majority of the cohort was postmenopausal (73.33%; 121/165).

### 3.2. Clinicopathology of the Cohort

The cohort had 86 (52.1%) clinically node negative (N0) cases and 79 (47.87%) clinically suspicious/node positive (N+) cases. The predominant molecular subtype was luminal with 128 out of 165 cases (77.57%) falling under this subtype. Unifocal tumors accounted for 82.42% (136/165) of the cohort, with the majority of the cohort comprising clinical tumor stages T1 (33.33%; 55/165) and T2 (56.96%; 94/165) and the pathological stage of IIA (38.18%; 63/165). IDC was the predominant tumor type in this cohort (86.66%, 143/165). Table 2 summarizes the detailed figures of the clinicopathological features of this cohort.

### 3.3. Representative Case Findings: This Representative Case Provides Insights into How the Nodes Were Distinguished as N0 in This Study Setting

**Figure 3:**
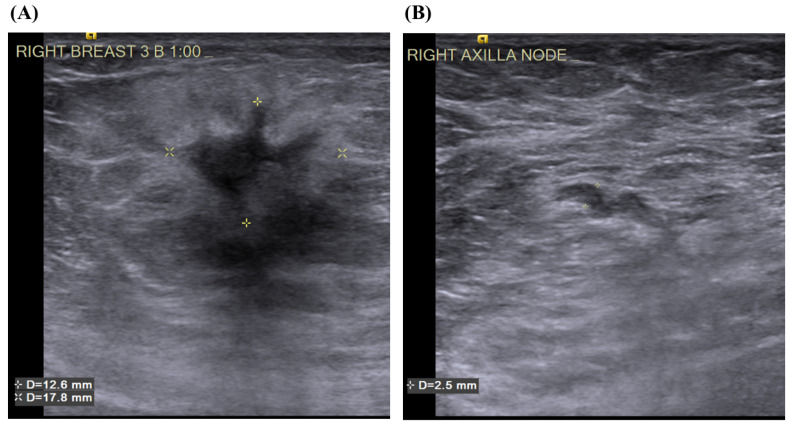
(Case 1): A representative example of a true negative case. A patient presented with a lump in her right breast measuring 17.8 × 12.6 mm at 12:00–1:00 position as seen in (**A**). A right axillary node visualized on PAUS as seen in (**B**) showed a cortical thickness < 3 mm with an intact fatty hilum, classifying it as N0 as per our criteria. Final HPE also proved all the 3 excised nodes to be free of metastasis, thus proving our PAUS result to be a true negative one.

In Figure 3 (Case 1), a 71-year-old patient presented with a lump in the right breast. (**A**) Right breast US images show a non-circumscribed hypoechoic mass with spiculations in the 12:00 o’clock–1:00 o’clock position, outer third, measuring 17.8 × 12.6 mm. (**B**) PAUS image shows a right axillary node with a thin cortex and an intact fatty hilum. The cortical thickness of 2.5 mm classifies the case as N0 and is proven to be negative on HPE as well. HPE gave a diagnosis of a Grade II, ER/PR-positive HER2-negative invasive mammary carcinoma with focal DCIS with all three excised nodes proven to be free of metastasis.

### 3.4. Correlation Studies for PAUS Findings

Bivariate analysis of PAUS findings revealed several significant factors distinguishing between N0 (negative nodes) and N+ (positive nodes) cohorts. Prognostic nodal features like cortical thickness (*p* < 0.001), shape of the node (*p* < 0.001), fatty hilum status (*p* < 0.0001) and nodal margins (*p* < 0.001) were found to be significant distinguishers for N0 and N+. Additionally, primary tumor size on imaging (*p* = 0.004) and histopathological features like lymphovascular invasion (LVI) (*p* < 0.0001) and extranodal extension (ENE) (*p* < 0.0001) also showed a significant difference between the N0 and N+ groups, with node positivity increasing with tumor size with the presence of LVI and ENE. Conversely, demographic characteristics such as menopausal status, age group, and surgery type, along with pathological features like histopathological type and histological grade, and molecular subtypes luminal, HER2 and Triple Negative Breast Cancer (TNBC), did not show a statistically significant association with nodal status. Table 3 gives the detailed numbers and the significance values for pathological and PAUS parameters with PAUS findings.

### 3.5. Diagnostic Performance of PAUS

The diagnostic performance of PAUS, summarized in Table 4, demonstrated an accuracy of 90.69% to detect negative axillae with 78 of 86 N0 being true negatives. For the full cohort of 165, a sensitivity of 86.20% and a specificity of 71.02% was observed. The positive predictive value (PPV) was 61.72%, while the NPV was 90.47%, with a false-negative rate of <10%. The overall accuracy of PAUS was 76.36%. The positive likelihood ratio (LR+) was 2.9755 and the negative likelihood ratio (LR-) was 0.1941.

### 3.6. Diagnostic Performance of PAUS for SLNB Omission Criteria Defined by the SOUND [8] and INSEMA Trials [10]

The total cases in this study’s cohort according to SOUND and INSEMA trial criteria were (n) = 24, True negatives = 23, False negatives = 1 and Accuracy = 95.83%.

## 4. Discussion

The management of axillary lymph nodes in breast cancer has traditionally relied on surgical staging methods, primarily through SLNB or in cases of advanced disease, ALND. Preoperative nodal assessment typically includes clinical examination and imaging, along with fine-needle aspiration cytology (FNAC) or core needle biopsy of suspicious axillary nodes or SLNB to confirm nodal involvement. However, the dynamic landscape of breast cancer surgical management has been predominantly moving towards personalized and de-escalated strategies, with PAUS posing as a reliable, non-invasive method to accurately assess the status of axillary nodes and make informed surgical decisions. This study presents supportive evidence for the diagnostic performance of PAUS, for its ability to identify patients without axillary nodal involvement (N0). The findings suggest that PAUS may help identify subsets of patients in whom SLNB could potentially be omitted.

In this retrospective study at our centre, PAUS showed a high accuracy of 90.69% in identifying node-negative axillae and a sensitivity of 86.20%. These findings suggest that, when performed with stringent sonographic criteria and interpreted by trained breast radiologists, PAUS has the potential to reliably classify node-negative patients who can skip SLNB. At our centre, SLNB is routinely performed for all patients irrespective of their PAUS findings; hence, this study had a robust histopathological reference standard, thus strengthening the internal validity of these observations.

The bivariate analysis reinforced the established nodal features observed on PAUS that are statistically significant in predicting the axillary node status [11]. The shape, fatty hilum and margins of the lymph node (*p* < 0.0001) were identified as statistically significant distinguishing features between the N0 and N+ cohorts. Moreover, the size of the primary tumor observed on imaging showed a significant association with nodal involvement (*p* = 0.004), where the larger the size, the greater the probability of having a positive axillary nodal status on PAUS. Additionally histopathological features like LVI (*p* < 0.0001) and ENE (*p* < 0.0001) were seen to be statistically significant in differentiating between N0 and N+ cohorts. In contrast, conventional demographic factors, histopathological features, and receptor statuses (ER, PR, HER2) and molecular subtypes did not show a statistically significant correlation (*p* > 0.05). These findings support the idea of PAUS being promising in providing direct morphological assessment of nodal disease that can be complementary to biological assessment parameters rather than solely relying on them.

A clinically relevant strength of this study is the observed high NPV of PAUS in identifying true negative axillary nodes. Although the overall accuracy dropped with the inclusion of N+ patients, this reduction is the result of an intentional cautious threshold followed by our centre where even minimal sonographic suspicion is flagged and triaged to surgical staging. Hence, in this context, PAUS may serve as an effective triaging tool for SLNB, particularly in select low-risk patient populations.

The classification of cases as N0 using PAUS, based on our centre’s criteria, is exemplified by the case (Figure 3, Case 1) presented in the Results section. Using our centre’s criteria for N0 classification, i.e., an intact fatty hilum, cortical thickness <3 mm, and circumscribed margins, the node was correctly classified as negative. The PAUS assessment was proven correct by the final histopathology, highlighting the aptness of the adopted criteria at our centre in ruling out metastatic involvement.

This study also aligns well with defining trials assessing PAUS directly or indirectly for its capacity to identify patient cohorts that can safely forgo SLNB by validated criteria and those suggesting de-escalation strategies and less-invasive clinical decisions.

The ACOSOG Z0011 [7] trial has redefined breast cancer surgery by demonstrating that SLNB alone is non-inferior to ALND for patients with limited sentinel node metastases (1–2 positive nodes) who are undergoing breast-conserving surgery and adjuvant systemic therapy (AST). Its significant findings on reducing the extent to axillary surgery highlight the need of accurately identify patients who can safely forgo invasive surgical procedures. Although the Z0011 [7] trial did not directly assess PAUS, the current study’s ability to identify N0 patients through PAUS positively supports its significance in early and less invasive decision-making.

Resonating with the rationales of the Z0011 [7], SOUND [8] and INSEMA [10] trials discussed previously, the current study presents affirmative results supporting the safe omission of SLNB in patients accurately identified by PAUS as having no nodal burden (N0) and fitting the omission criteria defined by these trials. According to the ASCO guidelines [12], which were included by the SOUND [8] and INSEMA [10] trials as well, the current study demonstrates a high accuracy of 95.83%, providing a significant corroboration from a single institute in India for accurately identifying these patients preoperatively. The accuracy of the identification of negative axillae and overall NPV of PAUS observed in the current study also reaffirms the SLNB omission criteria laid down by the SOUND [8] and INSEMA [10] trials. Future large, multicentric cohort studies can further validate the ability of PAUS to identify the select patient population.

Several recent studies similarly assessing the efficacy of PAUS have shown promising results for the detection of negative axillae. Table 5 compiles the reported diagnostic performance of PAUS in recent studies along with the current study figures.

The relevance of these findings is particularly pronounced in the Indian context where variability in resources, access to nuclear medicine facilities and radiological expertise can limit uniform implementation of SLNB [3]. We present this retrospective study as a single-centre experience of PAUS, but moving ahead PAUS can function as a dependable, accessible and non-invasive modality for axillary assessment in such resource-constrained settings with the help of large, multicentric studies to validate the technique’s capacity and applicability.

Despite the promising findings, this study has several limitations that require consideration. First, its retrospective study design and single-institution setting may introduce selection and institutional bias, which may prevent the results being generalized to broader populations. Second, the study did not formally assess inter-observer variability in ultrasound interpretation, which could affect the reproducibility of the findings. Third, the NPV of PAUS is inherently dependent on disease prevalence, which may limit its applicability across different clinical settings. Lastly, the relatively small sample size and lack of long-term follow-up for recurrence or survival outcomes restrict the ability to draw definitive conclusions about the prognostic impact of omitting SLNB based solely on PAUS findings.

As a preliminary exploration, this retrospective analysis has demonstrated the feasibility and effectiveness of PAUS for node negative patients in this study’s institutional setting, serving as an important starting point for future prospective studies in select patient cohorts. Moving forward, prospective, large-cohort studies are warranted to validate these findings and to formally establish PAUS as a reliable tool for axillary assessment in breast cancer management.

The diagnostic capabilities of PAUS can be enhanced with the incorporation of additional US parameters like lymph node vascularity assessment via Doppler US and elastographic parameters. Moreover, integrating complementary imaging modalities like Contrast-Enhanced Ultrasound (CEUS), Contrast-Enhanced Mammography (CEM) and Magnetic Resonance Imaging (MRI) can facilitate a holistic understanding of axillary disease and further guide clinical decision-making. To promote standardization and facilitate the integration of PAUS into standard clinical practice, the establishment of a globally accepted scoring system is necessary. Such a standardized framework would enhance inter-observer reproducibility and consistency in the diagnostic process. The incorporation of artificial intelligence (AI) offers a promising avenue for advancing the understanding of the PAUS findings. AI-driven algorithms have the potential to optimize and automate the analysis of US findings, thus significantly improving the diagnostic accuracy of PAUS, particularly in resource-limited settings, such as in India. This technological integration could lead to more precise axillary staging and the avoidance of unnecessary surgical interventions ultimately leading to betterment in patient care. The integration of PAUS into the evolving domain of breast cancer management holds the potential for developing more personalized treatment strategies. By aligning technological advancements with clinical expertise, one can aspire to optimize outcomes for patients, marking a significant milestone in the trajectory of oncological care.

## 5. Conclusions

This retrospective analysis examines the feasibility of PAUS in accurately identifying node-negative axillae within this study’s institutional setting in India. The findings indicate that PAUS shows promise as a cost-effective [21], reliable tool for assessing axillary status, particularly in resource-limited environments where avoiding additional surgical procedures can significantly reduce patient burden and healthcare costs. While the study suggests good diagnostic accuracy in identifying patients without nodal involvement, these results remain exploratory given the patient numbers, operator-dependent nature of ultrasound and the retrospective design of the analysis. In the Indian context where healthcare economics play a critical role in treatment planning, the selective use of PAUS to safely omit SLNB in select patients could offer meaningful advantages. This approach aligns with the global trend toward de-escalation of axillary surgery, while emphasizing affordability and accessibility. Looking ahead, prospective studies with larger cohorts are needed to validate these initial findings and to assess the role of PAUS even in post-NAST settings. Developing standardized PAUS criteria and integrating it with complementary imaging modalities could further enhance its diagnostic precision and facilitate its broader implementation in LMIC healthcare systems like India’s.

## Figures and Tables

**Table 1:** Demographic features of the cohort.

Feature	Class	No. (*n* = 165)	Percentage (%)
Age (years)	Median	60 (27–84)	-
	≤40	11	6.66
	41–60	78	47.27
	>60	76	46.06
Comorbidities	Yes	90	54.54
	No	75	45.45
Menopausal status	Pre	44	26.66
	Post	121	73.33

**Table 2:** Clinicopathological features of breast cancer patients in the cohort (NA (not applicable) include DCIS and papillary tumors with no grade of Bx; HER2-positive group includes all HER2+ tumors (irrespective of hormone receptor status).

Feature	Class	No. (*n* = 165) (ALNs/Biopsies Assessed)	Percentage (%)
Molecular subtypes	ER/PR	128	77.57
	HER2+	14	8.48
	TNBC	23	13.93
Focality	Unifocal	136	82.42
	Multifocal/Multicentric	29	17.57
Clinical Tumor (cT)	Tis	14	8.48
	T1	55	33.33
	T2	94	56.96
	T3	2	1.21
Clinical Node (cN) positivity	N0 (negative node)	86	52.12
	N+ (positive node)	79	47.87
Pathological tumor stage (Grouped)	Stage 0	10	6.06
	Stage IA	47	28.48
	Stage IB	0	0
	Stage IIA	63	38.18
	Stage IIB	30	18.18
	Stage IIIA	12	7.27
	Stage IIIB	0	0
	Stage IIIC	3	1.81
Biopsy tumor grade	I	7	4.24
	II	110	66.66
	III	24	14.54
	NA	24	14.54
Type of tumor	IDC	143	86.66
	DCIS	14	8.48
	ILC	8	4.84
Surgery type	BCS	139	84.24
	MRM	26	15.75

**Table 3:** Correlation of cohort features with PAUS findings (*—significant).

Type	Characteristics		PAUS Results		*p*-Value
			N0	N+	
Histopathological features	Extranodal extension (ENE)	Absent	85	60	* 0.00001
		Present	1	19	
	Lymphovascular invasion (LVI)	Absent	80	55	* 0.000099
		Present	6	24	
PAUS features	Shape of the node	Oval	85	56	* 0.00001
		Round	1	23	
	Fatty hilum	Intact	86	20	* <0.0001
		Displaced/Lost	0	59	
	Node margins	Regular	86	29	* <0.0001
		Irregular	0	50	
	Imaging primary tumor size (mm)	<20	34	14	* 0.003991
		20–50	49	64	
		>50	3	1	
	Cortical thickness (mm)	<3	84	2	* <0.0001
		3–4	1	24	
		>4	1	53	

**Table 4:** Diagnostic performance of PAUS.

Statistic	Value	95% CI
Accuracy for detecting N0	90.69%	84.55–96.33%
Sensitivity	86.20%	77.33–95.08%
Specificity	71.02%	62.43–79.62%
PPV	61.72%	51.14–72.31%
NPV	90.47%	84.19–96.75%
Accuracy	76.36%	69.88–82.84%
LR+	2.9755	2.5384–3.4878
LR-	0.1941	0.1410–0.2673

**Table 5:** Comparison of the performance of PAUS reported in recent studies with the current study.

Study	Sample Size	Sensitivity (%)	Specificity (%)	PPV (%)	NPV (%)	Accuracy (%)
Rukanskienė et al. [13]	227	52.9	89.6	47.4	91.5	84.1
Vidallé et al. [14]	530	67.1	97.9	92.7	88.1	89.1
Liu et al. [15]	3944	90.4	68.2	36.5	97.2	71.9
Saltarin et al. [16]	620	40.8	92.7	75.8	73.6	74
Jamaris et al. [17]	383	45.5	80.7	76.5	51.8	60.3
Radosa et al. [18]	2059	79	100	100	93	94
Olfatbakhsh et al. [19]	140	56	88	75	76	76
Stephens et al. [20]	263	23	94	37	88	-
Current study	165	86.2	71.02	61.72	90.47	76.36

## Data Availability

The data presented in this study are available on request from the corresponding author due to ethical reasons.

## Abbreviations

List of abbreviations:

**Abbreviation**	**Full form**
ACOSOG	American College Of Surgeons Oncology Group
ALN	Axillary Lymph Node
ALND	Axillary Lymph Node Dissection
ASCO	American Society of Clinical Oncology
AST	Adjuvant Systemic Therapy
BC	Breast Cancer
BCS	Breast Conservation Surgery
CEUS	Contrast Enhanced Ultrasound
CEM	Contrast Enhanced Mammography
DCIS	Ductal Carcinoma In Situ
ENE	Extra-Nodal Extension
ER	Estrogen Receptor
ESBC	Early Stage Breast Cancer
FNAC	Fine Needle Aspiration Cytology
HER2	Human Epidermal Growth Factor Receptor 2
HPE	Histo-Pathological Examination
IDC	Invasive Ductal Carcinoma
ILC	Invasive Lobular Carcinoma
LCIS	Lobular Carcinoma In Situ
LR	Likelihood Ratio
LVI	Lympho-Vascular Invasion
MRI	Magnetic Resonance Imaging
MRM	Modified Radical Mastectomy
NACT	Neo-Adjuvant Chemotherapy
NAST	Neo-Adjuvant Systemic Therapy
NPV	Negative Predictive Value
NST	No Special Type
PAUS	Preoperative Axillary Ultrasound
PPV	Positive Predictive Value
PR	Progesterone Receptor
SLNB	Sentinel Lymph Node Biopsy
SOUND	Sentinel node vs. Observation after axillary Ultrasound
TNBC	Triple Negative Breast Cancer
US	Ultrasound
